# Fatigue in Patients on Chronic Hemodialysis: The Role of Indoleamine 2,3-Dioxygenase (IDO) Activity, Interleukin-6, and Muscularity

**DOI:** 10.3390/nu15040876

**Published:** 2023-02-09

**Authors:** Alessio Molfino, Giovanni Imbimbo, Maria Ida Amabile, Thomas Ammann, Luana Lionetto, Gerardo Salerno, Maurizio Simmaco, Maria Grazia Chiappini, Maurizio Muscaritoli

**Affiliations:** 1 Department of Translational and Precision Medicine, Sapienza University of Rome, 00185 Rome, Italy; 2 Department of Surgical Sciences, Sapienza University of Rome, 00161 Rome, Italy; 3 Hemodialysis Unit, Fatebenefratelli Hospital, 00186 Rome, Italy; 4 Analytical Laboratory Unit, Sant’Andrea Hospital, Department NESMOS, Sapienza University of Rome, 00189 Rome, Italy

**Keywords:** fatigue, dialysis, Kyn/Trp ratio, IDO, body composition, inflammation, muscle mass

## Abstract

Fatigue is a frequent symptom in hemodialysis (HD), and the indolamine-2,3-dioxygenase (IDO) metabolic trap has been hypothesized in the pathogenesis of fatigue. The association between IDO activity according to fatigue and its relationship with muscle mass and function in HD patients was verified. Chronic HD patients were considered, and fatigue was assessed. The plasma kynurenines and tryptophan ratio (Kyn/Trp), as surrogate of IDO activity, and interleukin (IL)-6 were measured. Muscularity was assessed by BIA and muscle strength by hand-grip dynamometer. 50 HD patients were enrolled, and fatigue was present in 24% of the cohort. Patients with fatigue showed higher Kyn/Trp (*p* = 0.005), were older (*p* = 0.007), and IL-6 levels resulted higher than in non-fatigue patients (*p* < 0.001). HD patients with fatigue showed lower intracellular water (surrogate of muscle mass) (*p* < 0.001), as well as lower hand grip strength (*p* = 0.02). The Kyn/Trp ratio positively correlated with IL-6 and ECW/ICW (*p* = 0.004 and *p* = 0.014). By logistic regression analysis, higher ICW/h^2^ was associated with lower odds of fatigue (OR, 0.10; 95% CI, 0.01 to 0.73). In conclusion, our cohort fatigue was associated with a higher Kyn/Trp ratio, indicating a modulation of IDO activity. The Kyn/Trp ratio correlated with IL-6, suggesting a potential role of IDO and inflammation in inducing fatigue and changes in muscularity.

## 1. Introduction

Fatigue is a frequent complaint of patients with chronic kidney disease (CKD), especially by those maintained on hemodialysis (HD) [1], being present in 42–89% of the patients, depending on the type of the cohort studied [2]. Fatigue is a debilitating symptom that negatively affects quality of life and prognosis in the setting of HD [2,3]. Several factors contribute to fatigue, including depression, anemia, malnutrition, and increased circulating inflammatory markers [2]. Some of the contributing factors that are often implicated in the development of fatigue include impaired oxygen delivery and consequent lactic acidosis, conditions that are strictly associated with an altered skeletal muscle function [1,2]. Metabolic acidosis, immobilization, obstructive sleep apnea, and several drugs were also described as important players in the pathophysiology of fatigue [1]. Importantly, inflammation plays a crucial role in inducing many metabolic alterations in HD [4] and likely contributes to fatigue due to the interaction of proinflammatory cytokines and the central nervous system, also promoting anorexia and peripheral tissue changes, mainly represented by enhanced muscle and adipose tissue catabolism [2,5,6].

Different factors sustain the inflammatory processes in renal patients. In fact, the enhanced release of proinflammatory cytokines [6,7,8], through the activation of macrophages and monocytes, represents one of the main mechanisms underlying the metabolic-inflammatory response. This phenotype is associated with an increased mortality, especially for cardiovascular events [2,7,8]. Moreover, this altered inflammatory profile often characterizes the malnutrition/inflammation/cachexia syndrome [4]. In patients with renal impairment (not exclusively on HD), systemic inflammation is typically identified by an increase in low-grade inflammatory markers, and this clinical picture also contributes to the worsening of the chronic nephropathy. In fact, inflammation is also associated with altered calcium-phosphorus metabolism and increased oxidative stress [6,9].

Interestingly, increased IL-6 circulating levels may activate indoleamine 2,3-dioxygenase (IDO), the pathway involved in many catabolic processes, determining the degradation of tryptophan producing kynurenine [7]. For this reason, the Kyn/Trp ratio may represent a surrogate of the IDO activity [7]. Schefold et al., in a cohort of patients with moderate to severe CKD, observed that IDO activity increased in association with the severity of the CKD (not influenced by hemodialysis treatment) and it positively correlated with markers of inflammation [8].

Also, kynurenine and tryptophan were investigated on their potential role on the central nervous system, inducing depression-like symptoms and anorexia [9,10], as well as in cancer-associated fatigue [10].

For this reason, in the present study, we assessed the association between IDO activity and fatigue in patients on chronic HD and their relationship with inflammation and muscle mass and function.

## 2. Materials and Methods

### 2.1. Study Design and Patient’s Characteristics

This is an observational, cross-sectional study on patients with end-stage renal disease (ESRD) from the HD Unit of “Fatebenefratelli” Hospital, Rome, Italy. Patients were on HD regimens three times a week for 4-h sessions.

We enrolled patients of age ≥18 years with a time on dialysis of at least 1 month and the capability to provide informed consent. Exclusion criteria were the presence of other chronic diseases possibly determining fatigue and malnutrition (e.g., severe chronic obstructive pulmonary disease, chronic heart failure, liver cirrhosis, cancer, etc.). The study was conducted according to the Declaration of Helsinki and approved by the local ethics committee. We obtained the written informed consent before the enrollment.

We collected clinical and demographic characteristics during the enrollment visit, including age, sex, weight, height, comorbidities, and time on dialysis. From the clinical records, we obtained data on biomarkers related to fatigue and inflammation, including hemoglobin, C-reactive protein, and albumin levels.

### 2.2. Evaluation of Fatigue, Body Composition, and Muscle Strength

All patients completed a validated questionnaire investigating the barriers to physical activity [11,12]. Patients were diagnosed with fatigue if they responded to having fatigue both during the dialysis and on non-dialysis days. This allowed us to consider patients with a severe grade of fatigue.

Patients performed bioimpedance analysis in a supine position (Model 101, single-frequency, Akern, Florence, Italy) to assess indices of muscularity 1 h after the end of the second HD session of the week, as previously described [12,13]. The electrodes were put on the hand and foot of the non-access side of the body to inject current, while electrodes placed on the wrist to the ankle were used to measure voltage. Total body water was estimated using the resistance extrapolated to frequency and parameters, including intracellular water (ICW), as surrogate of muscle mass [14], as previously shown [13].

Muscle strength was measured testing the handgrip strength with a handle dynamometer (JAMAR, Sammons Preston Rolyan, Bolingbrook, IL, USA) on the day of the enrollment visit before the HD session. The test consisted of 3 measurements with each hand. The mean of the attempts of the strongest hand was utilized as a measure of muscle strength [15].

### 2.3. Tryptophan and Kynurenine Assays by Liquid Chromatography–Tandem Mass Spectrometry (LC-MS/MS) Method

Blood samples were collected from the participants before the second HD session of the week and centrifuged obtaining serum samples and stored at −80 °C until analysis. Sixty microliters of serum were treated by 60 µL of deproteinizing solution. The mixture was mixed for 30 s and centrifuged at 14.000× *g* rpm for 15 min. 10 µL of clean supernatant was injected into the chromatographic system. Compounds were detected using a LC–MS/MS analytical method [16,17]. Chromatographic separation of analytes was made utilizing an Agilent Liquid Chromatography System series 1100 (Agilent Technologies, Santa Clara, CA, USA), on a F5 column (Phenomenex, Torrance, CA, USA) equipped with a security guard pre-column (Phenomenex, Torrance, CA, USA). The mobile phase included a solution of 0.1% aqueous formic acid and 100% acetonitrile; elution was realized at a flow rate of 300 μL/min, using an elution gradient, as previously described [17]. The mass spectrometry was realized on a 3200 triple quadrupole system (Applied Biosystems, Foster City, CA, USA) supplied with a Turbo Ion Spray source, and the detector was set in the positive ion mode, as previously described [17]. The instrument was set in the Multiple Reaction Monitoring (MRM) mode. Data were collected and analyzed by the Analyst 1.5.1 Software.

### 2.4. Serum Interleukin (IL)-6 Assay

IL-6 serum concentrations were assessed by the ELISA technique, determining the formation of the antibody-antigen complex in a single step in 90 min. The procedure consisted in serum and antibody mixing, incubation, washing, and adding the final substrate. All the samples were tested by the available kits (IL-6 ELISA KIT, Abcam ab178013) according to the instructions available from the manufacturer.

### 2.5. Statistical Analyses

Patient characteristics were shown as mean (±SD) and median (IQR) for normally and non-normally distributed variables, respectively. Dichotomous variables were shown as a number (%). T-Test and Mann–Whitney U tests were used to study the differences among patients with and without fatigue for a continuous variable according to the normal distribution, as appropriate. A chi-squared test was used for categorical variables. The Pearson correlation coefficient was implemented to ascertain the correlation between continuous variables. Due to the non-normal distribution of IL-6 values, we used the natural logarithm for the correlation analysis. Finally, we performed a logistic regression analysis to examine factors associated with fatigue. We used a model that included the Kyn/Trp ratio as a surrogate of IDO activity, with ICW normalized for height (h) (ICW/h^2^), as proxy of muscularity [14], and IL-6 as a measure of inflammation; these variables were adjusted for sex, BMI, and age. For all the analyses, a *p*-value < 0.05 was considered statistically significant.

## 3. Results

### 3.1. Patient’s Characteristics

We enrolled 50 patients affected by ESRD on chronic HD (37 males, 74%) with a mean age of 66.3 ± 15.6 years, a median time on dialysis of 33 months (13; 91) and a median BMI of 23.5 kg/m^2^ (22; 27).

In our cohort, fatigue on both dialysis and non-dialysis days was present in 12 patients (24%). Patient’s characteristics based on the presence/absence of fatigue are summarized in Table 1. In summary, patients with fatigue were older than those without fatigue, whereas no differences were present in sex, BMI, hemoglobin, and C-reactive protein levels (Table 1).

### 3.2. Fatigue and Indices of Muscle Mass and Function

Patients with fatigue presented with a reduced ICW/h^2^ (L/m^2^) with respect to those without fatigue (4.73 ± 1.65 vs. 6.61 ± 1.67; *p* = 0.001), and ECW/ICW was increased in fatigue patients (1.66, IQR 1.27; 1.81 vs. 0.95, IQR 0.84; 1.09, *p* = 0.0003).

Muscle function assessed by handgrip strength (mmHg) was decreased in patients with fatigue (18.73 ± 5.04) with respect to those without fatigue (26.31 ± 10.09) (*p* = 0.02).

### 3.3. Association between Fatigue and Kyn/Trp Ratio and IL-6 Serum Concentrations

**Figure 1:**
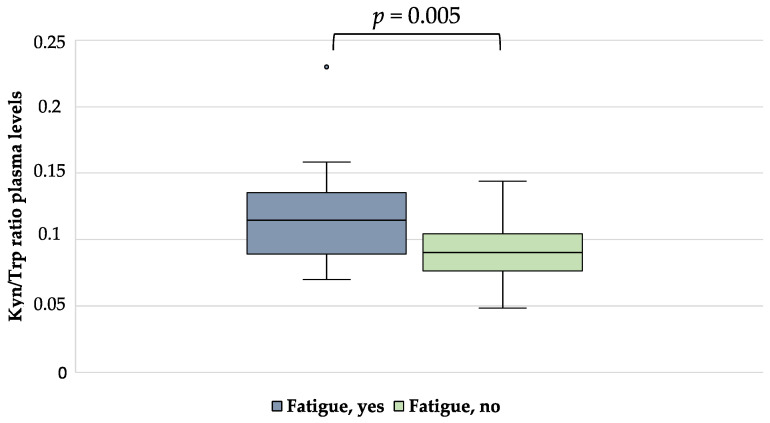
Kyn/Trp ratio in plasma of HD patients with and without fatigue.

Patients with fatigue presented with an increased Kyn/Trp ratio compared to non-fatigue patients (0.12 ± 0.04 vs. 0.09 ± 0.02, *p* = 0.005) (Figure 1). No significant differences were seen in terms of kynurenine plasma levels and tryptophan concentration between fatigue and non-fatigue patients (*p* = 0.806 and *p* = 0.104).

**Figure 2:**
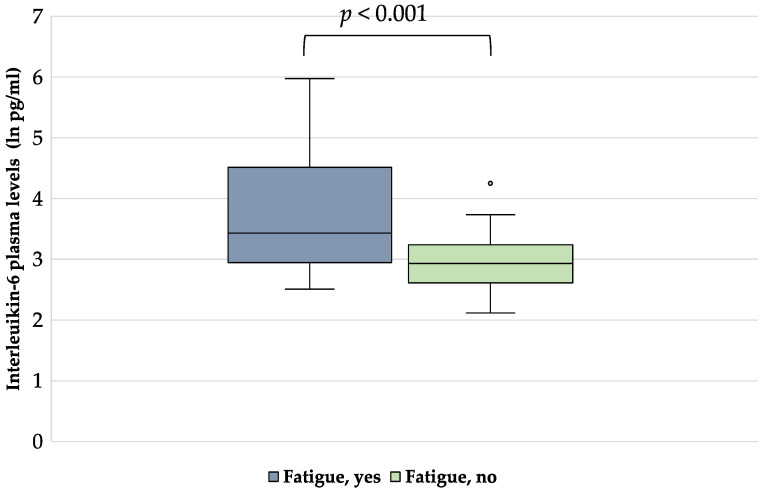
Interleukin-6 levels in plasma of HD patients with and without fatigue.

Moreover, IL-6 levels (ln) (pg/mL) resulted higher in patients with fatigue with respect to those without fatigue (3.70 ± 0.99 vs. 2.95 ± 0.47, *p* < 0.001) (Figure 2).

### 3.4. Correlation between ICW, IDO Activity, and IL-6 Concentration

When considering the entire cohort of HD patients, ECW/ICW positively correlated with the Kyn/Trp ratio (r = 0.35; *p* = 0.01) and with IL-6 (r = 0.40; *p* = 0.004). IL-6 positively correlated with the Kyn/Trp ratio (r = 0.40; *p* = 0.004).

### 3.5. Regression Analysis of Factors Associated with Fatigue

To examine the clinical factors associated with fatigue, we performed a linear regression analysis, including in the model Kyn/Trp, ICW/h^2^, as proxy of muscularity, and IL-6, adjusted for sex, BMI, and age. We observed that higher levels of ICW/h^2^ were significantly associated with a reduced risk of fatigue (OR 0.100; 95% CI 0.014; 0.731; *p* = 0.02) (Table 2).

## 4. Discussion

In our cohort, we found that fatigue was present in about one-quarter of HD patients. This observation is in line with those obtained in other studies where the presence of severe symptoms of fatigue were diagnosed in 25% of the renal population [1], although in other cohorts, the prevalence was much higher [1] and assessed by different instruments mainly based on questionnaires (Table 3).

Interestingly, we specifically investigated the presence or absence of fatigue during dialysis and non-dialysis days. In fact, data available in the literature are not so clear regarding potential differences between the severity of the symptoms between dialysis and non-dialysis days. However, many factors may determine variations of fatigue symptoms during the week, including lifestyle and psychological issues that can be more present during dialysis days [2]. In this light, patients are generally inclined to be more active on non-dialysis days, likely because there is more time available to exercise during those days.

Fatigue represents one of the most commonly diagnosed symptoms in patients with CKD, and its prevalence increases according to the negative evolution of the underlying renal condition [1]. Data on fatigue epidemiology are not univocal considering that its variability in terms of prevalence depends on the type of population studied and on the tools used for the diagnosis [18,19]. Fatigue is a distressing symptom and can be associated with physical inactivity, as well as with depression [19]. The highest prevalence of fatigue is found in CKD patients at stage 5, and, in particular, when receiving dialysis treatment [1]. The results obtained in different studies regarding the association between fatigue and depression in dialysis patients are not univocal [18,19]. This is determined by the fact that very often HD patients are not routinely screened for depression; therefore, it is not possible to clearly estimate its prevalence in accordance with the presence of fatigue. Of note, depression as well as fatigue are factors known to be associated with poor outcomes, such as reduced survival and increased hospitalization [20].

In our study, patients with fatigue were older than those without, whereas no differences in time on dialysis and BMI, as well as protein catabolic rate, were detected. Fatigue is a condition known to negatively influence nutritional status and, in particular, body composition [1]. On one hand, the presence of fatigue reduces the chance to be physically active and therefore negatively affects muscle mass [2]. On the other hand, low muscularity, determined by several catabolic factors associated with HD treatment, may exacerbate the onset and the progression of fatigue [4]. Although fatigue can be diagnosed through different tools [1], a reliable biomarker of this condition is not yet available.

In other clinical settings, fatigue was associated with a specific amino acid imbalance [21], and uremia is known to be characterized by an alteration in protein and amino acid metabolism, playing crucial roles either directly or by the production of toxins, with a resultant negative nitrogen balance, muscle atrophy, reduced protein synthesis, and abnormal intracellular free amino acid concentrations, including tryptophan plasma levels [21]. In particular, tryptophan is an essential amino acid that must be supplied through the diet, and most of its free concentration is metabolized to form kynurenines by the two rate-limiting enzymes IDO and tryptophan 2,3-dioxygenase (TDO) [7]. Interestingly, recent data indicate that kynurenine metabolism, including circulating levels of tryptophan, kynurenine, kynurenic acid (KA), and other metabolites, is dysregulated in chronic fatigue syndrome [22]. Experimental evidence showed that the kynurenine pathway is implicated in fatigue mediated by the central nervous system [23]. In fact, Yamamoto et al. found that the direct administration of kynurenic acid into the central nervous system of rats promoted a significant reduction in physical activity, and kynurenic acid determined a dose-dependent worsening of fatigue induced by physical activity [24].

In line with these available data, in our cohort of HD patients, we observed a higher Kyn/Trp ratio in patients with fatigue with respect to those without fatigue, suggesting an increased IDO activity. In particular, IDO was observed to be increased in patients with CKD with respect to the healthy population [25]. In fact, Malhotra et al. described in HD patients higher circulating kynurenine levels in association with depression and fatigue; however, no association was found between Kyn/Trp levels and fatigue [25], which is different from what we observed in our cohort. This may be at least in part caused by the different tools used in the two studies to assess fatigue and to the different severity of fatigue among the two cohorts, considering that Malhotra et al. included patients with mild to moderate fatigue, and we enrolled patients presenting with fatigue on both dialysis and non-dialysis days (very likely with greater fatigue) [25]. Noteworthy, we observed a positive correlation between IL-6 levels and the Kyn/Trp ratio, suggesting a relationship between inflammation and IDO activity. In this regard, the Kyn/Trp ratio was related with markers of inflammatory status and with molecules of acute phase response, consistent with the activation of IDO in innate immune cells [26] and, in the setting of hemodialysis, an association between the Kyn/Trp ratio and IL-6 was also described when depression was present [27].

Fatigue is frequently present when decreased muscularity and low muscle function are diagnosed among CKD patients, including those chronically maintained in HD [1]. In our cohort, we found a significant reduction in both muscle mass and muscle function in patients with fatigue and, interestingly, experimental data indicate that tryptophan metabolism is involved in the process of muscle atrophy [28].

By our regression analysis, including in the model the Kyn/Trp ratio, IL-6, and ICW/h^2^ (proxy of muscle mass), we observed a significant association only between fatigue and the level of muscularity. However, we cannot completely exclude the role of IDO and inflammation in the development of fatigue in HD considering the potential interplay of all these components in determining muscle atrophy as well as fatigue. Noteworthy, patients with fatigue are those with a median time on dialysis of 57 months compared to 26 months of those without fatigue. Although this data did not reach the statistical significance, the clinical negative impact of the duration of HD treatment on muscularity may interfere in our model with the significance of other factors associated with fatigue, including inflammation and IDO activity.

Additional conditions that should be considered when diagnosing and treating fatigue in HD patients are represented by the presence of altered serum phosphorus concentrations, hyperparathyroidism, the consequent osteodystrophy, and bone remodeling [1]. In fact, body composition changes in HD may affect not only muscle mass but also very frequently bone metabolism. In particular, HD patients, due to renal osteodystrophy, are at high risk of falls, fractures, and immobilization leading to a vicious circle worsening fatigue, muscle mass, physical performance, and quality of life [29].

The data obtained in the present investigation, if further confirmed, could allow the identification of kynurenines, as well as the Kyn/Trp ratio, as potential innovative molecular targets for the diagnosis and clinical management of fatigue associated with changes in muscularity in HD patients. Moreover, these patterns may have implications in the prognosis of patients on dialysis. Profiling the alterations in tryptophan metabolism may help to identify metabolic and nutritional changes and to support the possibility of developing a suitable personalized nutritional intervention aimed at ameliorating fatigue and body composition in end-stage renal disease. In this light, our results—specifically regarding the metabolic profiling of patients (IDO activity) with fatigue—should be considered as part of a personalized medicine program aimed at monitoring changes in biomarkers during or after metabolic and nutritional interventions.

Our study has some limitations, including the number of participants, representing a single-center investigation, and the cross-sectional design, which may limit the observation of the effect of IDO, fatigue, and changes in muscularity in a longer frame of time. The assessment of fatigue may be performed with more complete tools using a severity scale, considering that our questionnaire investigated the presence of fatigue on both dialysis and non-dialysis days, since these questions were reliable in CKD [1]. In our analyses, additional factors associated with fatigue were not evaluated, including the presence of osteodystrophy.

In conclusion, in our study, fatigue was associated with a higher Kyn/Trp ratio, indicating a modulation of IDO activity in this condition. This surrogate of IDO activity correlated with inflammatory markers, suggesting a potential role of IDO and IL-6 in inducing, and likely worsening, fatigue and changes in muscle mass and function among HD patients. Factors interfering with fatigue and IDO should be further better identified in HD patients, in particular to confirm the role of changes of muscularity on fatigue dependently or not of IDO activity.

## Figures and Tables

**Table 1:** Patient’s characteristics.

Parameter	Patients on Hemodialysis (*N* = 50)		*p*-Value
	Fatigue (*N* = 12)	No Fatigue (*N* = 38)	
Male, n (%)	8 (67)	29 (76)	0.506
Age, years	76.6 ± 11.7	63 ± 15.4	0.007
Body mass index, kg/m^2^	24.58 ± 3.06	24.82 ± 6.06	0.899
Time on dialysis, months	57 (38; 92)	26 (12; 79)	0.116
Protein catabolic rate, g/kg/d	0.87 ± 0.19	0.98 ± 0.27	0.237
Hemoglobin, g/dL	10.7 ± 1.7	11.6 ± 1.3	0.072
C-reactive protein, mg/dL	0.92 (0.22; 1.70)	0.32 (0.16; 0.67)	0.103
Albumin, g/dL	3.6 ± 0.3	3.7 ± 0.3	0.263
Creatinine, mg/dL	8.5 ± 2.0	9.7 ± 2.6	0.165
Diabetes, n (%)	5 (42)	8 (21)	0.298
Hypertension, n (%)	9 (75)	27 (71)	0.918
BIA parameters			
ICW, L	13.8 ± 2.0	18.4 ± 5.6	<0.001
ECW, L	22.2 ± 7.0	18.0 ± 5.2	0.03
ECW/ICW	1.66 (1.27; 1.82)	0.95 (0.84; 1.09)	<0.001
ICW/h^2^, L/m^2^	4.7 ± 1.7	6.6 ± 1.7	0.001
Hand grip strength, mmHg	18.7 ± 5.0	26.3 ± 10.1	0.02
Kynurenine, µg/mL	0.52 ± 0.24	0.51 ± 0.15	0.806
Tryptophane, µg/mL	4.7 ± 2.1	5.6 ± 1.5	0.104
Kyn/Trp ratio	0.12 ± 0.04	0.09 ± 0.02	0.005
Interleukin-6, pg/mL	30.9 (19.1; 80.9)	18.6 (14.0; 25.1)	0.011

Abbreviations: bioimpedance analysis, BIA; intracellular water, ICW; extracellular water, ECW, height, h; Kynurenine, Kyn; Tryptophan, Trp. Median (IQR) is shown for variables non-normally distributed.

**Table 2:** Association of fatigue with IL-6, Kyn/Trp ratio (IDO activity), and ICW/h^2^ (index of muscularity).

Parameters	OR (95% CI)	*p*-Value
IL-6 (pg/mL)	1.048 (0.991; 1.108)	0.101
Kyn/Trp ratio	1.317 (0.818; 2.120)	0.257
ICW/h^2^ (L/m^2^)	0.100 (0.014; 0.731)	0.023

Model adjusted for age, sex, and BMI; Abbreviations: interleukin-6, IL-6; kynurenine, Kyn; tryptophane, Trp; intracellular water, ICW; height, h.

**Table 3:** Summary of the characteristics of the main tools used for the assessment of fatigue in patients with chronic kidney diseases.

Instrument Used to Diagnose Fatigue	Number of Items Included	Scoring System	Time of Administration	Notes
FACIT-Fatigue	13 questions	5-point Likert-type scale for each item with a minimum score of 0 and maximum score of 52. Lower is the score, higher is the grade of fatigue	Less than 5 min to complete the entire questionnaire	The scale represents a quantitative measure of fatigue, and a cut-off of ≤44 was utilized to diagnose the presence of fatigue The scale was used in several chronic conditions, including cancer and chronic kidney disease
POSs	1 out of 17 questions identifying fatigue as “weakness or lack of energy”	5-point Likert-type scale (absent, mild, moderate, severe and overwhelming)	Less than 5 min to complete the entire questionnaire	The questionnaire was originally designed for patients on palliative care and then adapted for patients with chronic kidney disease
DSI	1 out of 17 questions identifying fatigue as “feeling tired or lack of energy”	5-point Likert-type scale (not at all, a little bit, some-what, quite a bit, and very much) ranging from 0 to 4	Less than 5 min to complete the entire questionnaire	The questionnaire was designed to investigate 30 symptoms among patients on dialysis
MSAS-SF	1 out of 32 items identifying fatigue as “lack of energy”	5-point Likert-type scale; 0 (not at all) to 4 (very much)	Less than 5 min to complete the entire questionnaire	The MSAS-SF include global distress index, the physical symptom distress score, the psychologic symptom distress score comprehending the evaluation of 6 psychological symptoms
ESAS-r	1 out of 10 items identifying fatigue as tiredness	Symptoms rated from 0 to 10	Less than 5 min to complete the entire questionnaire	It is a quantitative score used originally in palliative care settings and more recently to assess physical and psychological symptoms in patients with end-stage renal disease
QIDS-SR16	1 out of 16 items identifying fatigue as grade of energy/fatigability	Likert scale of 0 (no change in usual level of energy) to 3 (unable to carry out most of usual daily activities due to lack of energy)	From 5 to 10 min to complete the entire questionnaire	The QIDS-SR16 has 16 items based on the 9 symptom domains of major depressive disorder. The score ranges between 0 to 27
BDI	1 out of 21 items identifying fatigue as tiredness	Likert scale of 0 (no fatigue) to 3 (severe fatigue)	From 5 to 10 min to complete the entire questionnaire	The BDI accounts for cognitive/affective features of depression and somatic aspects such as sleep disturbance and health concerns)
SF-12 vitality scale	1 out of 12 items identifying fatigue as grade of energy	Six-point Likert scale for the question “did you have a lot of energy”?	Less than 5 min to complete the entire questionnaire	The SF-12 is a self-reported outcome measure which is often used for the evaluation of quality of life in different settings. The SF-12 is a short version of the SF-36
DPEBBS	2 out of 24 items evaluating tiredness and muscle fatigue	Binary variable (i.e., yes or no)	Less than 5 min to complete the entire questionnaire	It is a 24-item questionnaire used to evaluate the perceived benefits and barriers to exercise of the patients. The scale includes 24 items (12 items of exercise benefits and 12 items of exercise barriers) and 2 open questions
Questions regarding barriers to physical activity	2 out of 23 barriers are represented by fatigue on non-dialysis days and fatigue on dialysis days	Patients were asked if “never”, “sometimes”, “often”, or “always” experienced that barrier. ”never” classified as not having the barrier	Less than 5 min to complete the entire questionnaire	These questions investigate broad the following barriers to exercise: psychological barriers, physical barriers, lack of time, and presence of comorbidities.

Legend: Functional Assessment of Chronic Illness Therapy, FACIT; Chronic Kidney Disease—Symptom Burden Index, CKD-SBI; Patient Outcome Scale (symptom module), POSs; Dialysis Symptom Index, DSI; Memorial Symptom Assessment Scale Short Form, MSAS-SF; Edmonton Symptom Assessment Scale revised, ESAS-r; Quick Inventory of Depressive Symptomatology Self-Report, QIDS-SR16; Beck Depression Inventory, BDI; 12-Item Short Form Health Survey, SF-12; Dialysis Patient-perceived Exercise Benefits and Barriers Scale, DPEBBS.

## Data Availability

The datasets generated and/or analyzed during the current study are not publicly available because they are the property of the Institution. The data are available from the corresponding authors on reasonable request with prior authorization from the Institution.
